# Morphological traits – desiccation resistance – habitat characteristics: a possible key for distribution in woodlice (Isopoda, Oniscidea)

**DOI:** 10.3897/zookeys.801.23088

**Published:** 2018-12-03

**Authors:** Diána sonka, Katalin Halasy, Krisztina Buczkó, Elisabeth Hornung

**Affiliations:** 1 Department of Ecology, University of Veterinary Medicine Budapest, H-1077 Budapest, Rottenbiller St. 50., Hungary; 2 Department of Anatomy and Histology, University of Veterinary Medicine Budapest, H-1078 Budapest, István St. 2., Hungary; 3 Department of Botany, Hungarian Natural History Museum, H-1087 Budapest, Könyves Kálmán Blvd. 40., Hungary; 4 HAS Centre for Ecological Research, Danube Research Institute, H-1113 Budapest, Karolina St. 29., Hungary

**Keywords:** Eco-morphology, habitat preference, intrageneric comparison, mortality, sympatric species, water loss

## Abstract

Terrestrial isopods, as successful colonizers of land habitats, show a great variety in species distribution patterns on a global, continental, or regional scale. On a local, within-habitat level these patterns reflect the species’ tolerance limits and the presence of suitable hiding places (shelter sites, refugia). Humidity preference reflects a species’ capability for water retention which, in turn, depends on the integumental barrier. Desiccation resistance is a key feature in isopod survival under different environmental conditions. The present study shows a correlation between cuticle thickness and desiccation resistance under three relative humidity (RH) ranges (about 30, 75 and 100% RH) in nine species, relating these to the species’ differences in meso- and microhabitat choices. Habitat preferences are also associated with differences in cuticle surface morphology. The results support our hypothesis that species distribution and desiccation resistance are associated with particular cuticular morphological traits. Phylogenetic relations seem to be less important in desiccation resistance than cuticle thickness and external morphology.

## Introduction

Terrestrial isopods (Isopoda, Oniscidea) are successful colonizers of land habitats with over 3700 described species (Sfenthourakis and Taiti 2015). The group has developed various morphological, physiological, and behavioral traits to survive the challenges of terrestrial life (e.g., desiccation, respiration, and reproduction) (Edney 1954, Schmalfuss 1984, Hornung 2011).

The sclerotized cuticle is the main barrier between oniscidean individuals and their environment. The crustacean exoskeleton is composed of four layers: the epicuticle, the procuticle (exo- and endocuticle), and the membranous layer (Travis 1955, Compère 1990). The epicuticular layer is important in inhibiting water loss (Cloudsley-Thompson 1977). It is divided into sublayers: the cement layer, the surface coat, the waxy layer, and the inner epicuticle. The waxy layer located within the cuticulin layer is not homologous with the external wax layer of insects (Compère 1990). However, as in insects, the waxy layer of oniscideans probably acts as a waterproofing barrier which reduces the tegumental water loss in terrestrial environments (Hadley 1982). Hadley and Quinlan (1984) detected cuticular lipids in the mesic *Porcelliolaevis*, although the amount of lipid present was not effectively reducing the transcuticular water loss.

It is well-known that terrestrial crustaceans lose water more rapidly than most other land arthropods due to their tegumental transpiration (Edney 1951, 1954, Powers and Bliss 1983, Lillywhite and Maderson 1988, [Bibr B39][Bibr B10]). The rate of water loss is affected by the environmental temperature and humidity (Edney 1951, 1968). Nevertheless, terrestrial isopods can take up water directly (Hoese 1981) and from water vapour (Wright and Machin 1990, 1993), which allows rapid recovery of water loss. The high cuticular permeabilities might explain the capacity for fast replacement of evaporative water loss in woodlice species (Wright and Machin 1993). Moreover, they can obtain preformed and metabolic water from their food and imbibe water through their mouth, anus (Spencer and Edney 1954, Cloudsley-Thompson 1977, Drobne and Fajgelj 1993) and water conducting system (Horiguchi et al. 2007). Greenaway and Warburg (1998) measured the water-flux in 16 terrestrial isopod species originating either from mesic or xeric habitats. Flux data were used to predict threshold water activities for water vapor absorption. They showed a trend of decreasing permeability to water with increasing aridity of the habitat, but no significant differences in the changes of mean water fluxes were found.

In terrestrial isopods, the epicuticle forms several surface structures such as tubercles, micro-ridges, setae, tricorns, plaques and pits (Holdich and Lincoln 1974, Schmalfuss 1978, Powell and Halcrow 1982, Holdich 1984). Some of these are sensory structures (Powell and Halcrow 1982, Price and Holdich 1980, Holdich 1984). Several studies have shown that tricorn receptors are common in terrestrial woodlice species (Sutton 1969, Schmalfuss 1978, Holdich 1984, Fernandes et al. 2016) but are absent in aquatic Crustaceans (Holdich 1976, Schmalfuss 1978). Therefore, these structures are presumably playing a role in terrestrial adaptation. Tsuneo (1989) showed that tricorn-type sensilla can receive mechanical, gustatory, and olfactory stimuli. They might be important in the perception of humidity, too. According to Holdich (1984) setae are exteroreceptors. The various plaques (micro-scales) have diverse functions, e.g., to conduct water in the cuticle in the water capillary system (Schmalfuss 1978, Hoese 1981). Powell and Halcrow (1982) showed that micro-scales are a common feature in isopods from different habitats. The dorsal surface of the exoskeleton is adapted to the microhabitat type e.g., the diverse tubercles and micro-ridges may provide anti-adhesive potential for small, endogeic species (Schmalfuss 1978, 1984).

We assumed that differences in preferred habitat type correlate with differences in desiccation resistance. We hypothesized that a key mechanism by which selection has generated the increased resistance was by increased thickness of the cuticle. We explored connections among species distribution, desiccation resistance, and morphological traits focusing on the following questions:

(i) What is the relationship between distribution and desiccation stress?

(ii) What is the relationship between microhabitat desiccation stress and species’ desiccation resistance?

(iii) What is the relationship between desiccation resistance and cuticle morphological traits?

We hypothesized that there is a higher similarity among morphological traits in epigeic species with similar ecological needs (that is sharing the same habitat) than among closely related species living under quite different environmental conditions. To test these hypotheses, we measured interspecific and intrageneric desiccation resistance and compared exoskeleton properties in a selected group of species from Central and Eastern Europe. While numerous studies have compared desiccation resistance of terrestrial isopod species (Hadley and Warburg 1986, Davis 1989, Greenaway and Warburg 1998, Tsai et al. 1998, Dias et al. 2012), our study is the first to relate ecological tolerance to morphological traits of cuticle.

## Materials and methods

For interspecific comparison six surface-active isopod species were tested. The species belong to different families and/or genera occurring in the same habitat: *Armadillidiumvulgare* (Latreille, 1804), *Cylisticusconvexus* (De Geer, 1778), *Orthometoponplanum* (Budde-Lund, 1885), *Protracheoniscuspolitus* (C. Koch, 1841), *Porcellionidespruinosus* (Brandt, 1933), and *Trachelipusrathkii* (C. Koch, 1841). *Armadillidiumvulgare* and *T.rathkii* are among the most frequent terrestrial isopod species in Hungary. The generalist *A.vulgare* is a frequent and widely distributed species in diverse habitat types (Hornung et al. 2007, Vilisics and Hornung 2008, Farkas and Vilisics 2013). The Central- and Eastern European species, *P.politus* and *O.planum* are connected to native, undisturbed deciduous forests in Central Europe (Tuf and Tufová 2005, Mock et al. 2007). *Cylisticusconvexus* and *P.pruinosus* are synanthropic species, indicating strong human influence (Korsós et al. 2002). We also examined four species belonging to the *Armadillidium* genus, showing allopatric distribution patterns. Their tolerance limits against environmental conditions are different, too. *Armadillidiumzenckeri* Brandt, 1833 is a habitat specialist, living exclusively in European swamps and marshlands (Sallai 1993, Farkas and Vilisics 2006). The atlanto-mediterranean *Armadillidiumnasatum* Budde-Lund, 1885 is an introduced species in Hungary. It is known from botanical gardens and greenhouses (Farkas and Vadkerti 2002, Korsós et al. 2002, Hornung et al. 2007). The future outdoor spread and establishment of *A.nasatum* is expected as it is widely distributed in the wild in other regions (Schultz 1961, Hornung and Szlavecz 2003, Giurginca 2006). *Armadillidiumversicolor* Stein, 1859 is widespread in Southeastern Europe. In Hungary it is most abundant along rivers (e.g., along the Danube) and lake shores in the Transdanubian region and the Mátra Mountains (Farkas 2007, Vona-Túri and Szmatona-Túri 2012). It occurs also in drier habitats, although not in Hungary (Ferenţi et al. 2012).

### Sampling and habitat characteristics

The studied sympatric species (*A.vulgare*, *C.convexus*, *O.planum*, *P.politus*, *P.pruinosus*, *T.rathkii*) were hand collected in a deciduous forest of the Buda Mountains, next to the western part of Budapest, Hungary (at Solymár; 47°35.094'N, 18°57.164'E). Two mesohabitat types, different in humidity and temperature, within the sampling area were searched for woodlice: a valley (Alsó-Jegenye) along a stream (Paprikás patak) accompanied by a trail and an elevated area with an ancient deciduous forest (Felső-patak Hill). Individuals of *A.zenckeri* came from a marshland (Ócsa, Hungary; 47°17'39.5"N, 19°12'27.9"E), and the specimens were collected on the waterside or directly above the water, under the bark of wooden duck-boards. Specimens of *A.nasatum* originated from the tropical glass house of the Botanical Garden of Eötvös Loránd University (Budapest, Hungary; 47°29'05.3'N, 19°05'01.6"E) and specimens of *A.versicolor* were collected in the Margaret Island surrounded by the Danube river (Budapest, Hungary; 47°31'44.4"N, 19°03'06.5"E).

### Experimental design

The collected individuals were kept in the lab in plastic boxes containing moist soil and litter for 14 days to ensure acclimatization. The specimens of each species were kept in 100% relative air humidity (RH) overnight to standardize the initial experimental conditions. This procedure ensured that animals replenished any possible water deficit. The isopods were without food for 24 hours, meanwhile they defecated their gut content, so this did not affect subsequent changes in body mass (Dias et al. 2012).

Water loss rate and mortality were studied in three different RH values in glass desiccators: an extremely dry (~30%), a relatively dry (~75%) and a humid one, nearly 100%. The humidity levels were acquired using silica gel (RH <30%), saturated sodium-chloride (RH 75%) and water (RH 100%) (Winston and Bates 1960). The experimental setup did not allow air to circulate. All experiments took 6 hours. We measured 20 specimens per species individually to avoid the water loss decreasing effect of aggregation of specimens (Broly et al. 2014). In the case of *A.vulgare* different sets of individuals (N = 20 in each experiment) were tested for the inter- and intrageneric comparisons. We used both sexes based on the results of a previous study (Dias et al. 2012) where no differences were found between the desiccation resistances of sexes. At the start of the experiment, the fresh body mass of each individual was measured by an analytical balance (Sartorius AG, Göttingen, Germany). Specimens were re-weighed at the end of the 6-h experiment and the weight-loss of each individual was standardized by its body weight. At the end of each experiment individuals were checked for mortality. In the present study ‘desiccation resistance’ was defined as the rate of mass-specific water loss (g g^-1^).

### Microscopic methods

To reveal the characteristics of the exoskeleton, light microscopic investigations (LM) were applied. For this purpose, we fixed two intermolt adult specimens from each species in 4% paraformaldehyde for 7 days (we chose the greatest size category in the sampled population). The fixation was followed by rinsing in distilled water (3 × 1 h). We decalcified the tissues overnight in 8% ethylenediamine-tetraacetic acid disodium salt (EDTA). After the tissues became pliable they were dehydrated through an ascending series of ethanol (50% – 1 h, 70% – overnight, 80%, 2 × 90%, 2 × 96%, and 2 × 100% – 1 h). After dehydration, the samples were kept in xylene (2 × 1 h). Thereafter the samples were infiltrated with paraffin wax at 60 °C overnight, and embedded afterward. Histological sections (7 µm) were cut with a Reichert 2040 microtome and stained with Weighert’s hematoxylin-eosin (HE) and Periodic Acid-Schiff (PAS) reagent. With PAS reagent, we aimed to detect possible polysaccharides in tissues and on the integumental surface. The histological sections were studied and photographed with a Leica DM750 microscope.

The surface tergal ornaments were examined with a Hitachi S-2600N scanning electron microscope (SEM). For SEM we used alcohol preserved (70% ethanol) intermolt adult males and females from each studied species. The samples were dehydrated through an ascending series of ethanol (50% – 1 h, 70% – overnight, 80%, 2 × 90%, 2 × 96%, 2 × 100% – 1 h) and were air dried (Schmalfuss 1978). Specimens were attached to aluminum holders. Coating with gold-palladium was with a XC7620 Mini Sputter Coater.

### Statistical analysis

To quantify the thickness of the tergites, 100 measurements were taken for each species using light microscope (LM) cross-sections (2 specimens, 5 slides, 10 measurements/slide; Image J software) (Csonka et al. 2013). In the case of *A.vulgare* different sets of specimens (N=2 for each) were tested for the inter- and intrageneric comparisons. We performed a one-way ANOVA followed by a post-hoc Tukey-test to compare cuticle thickness and water loss rates using R 3.2.3 software (‘Rcmdr’ Package). The assumptions of ANOVA were tested.

The relationship between mass-specific water loss, initial weight, and cuticle thickness was tested by Pearson correlation analysis (R 3.2.3 software). We analyzed the relationship between mass-specific water loss and the thickness of the epi- and procuticle separately. We used one-way ANOVA to analyze whether the variation in desiccation resistance as a function of cuticle thickness differs in the inter- and intrageneric groups (R 3.2.3 software). The comparison of the two groups was made based on the F value of the individual experiment’s ANOVA tests. An alpha value of p = 0.01 was used throughout.

## Results

### Desiccation resistance

**Figure 1.**
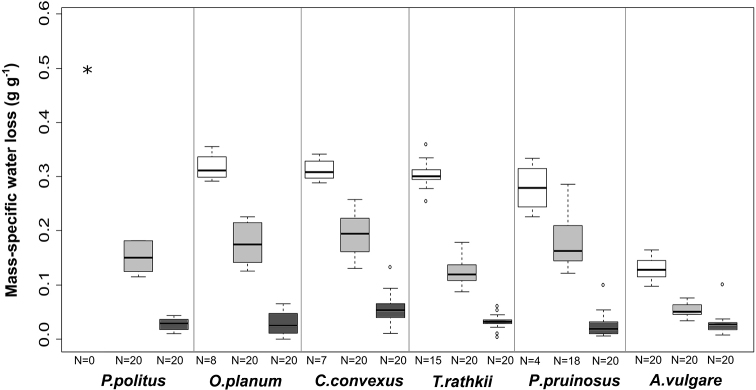
Mass-specific water loss of the survived individuals (*all individuals died) at three different relative humidity values (white: ~30%, medium gray: ~75%, dark grey: ~100%). The experiment took 6 hours. [Measures: median ± first quartile and max/min; species initials: *P.politus* – *Protracheoniscuspolitus*, *O.planum* – *Orthometoponplanum*, *C.convexus* – *Cylisticusconvexus*, *T.rathkii* – *Trachelipusrathkii, P.pruinosus* – *Porcellionidespruinosus*, *A.vulgare* – *Armadillidiumvulgare*].

**Figure 2.**
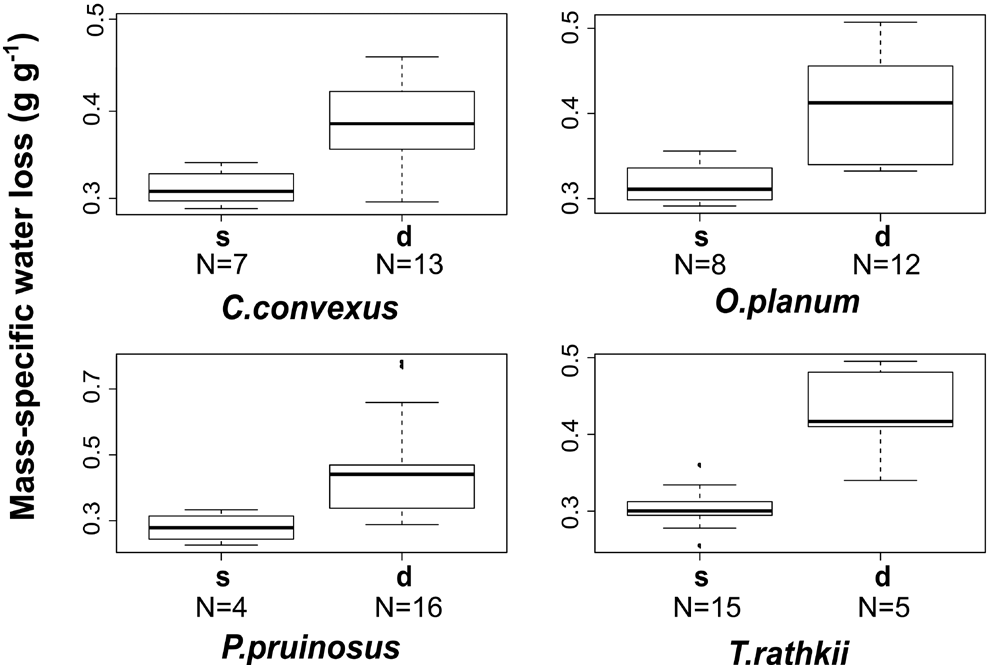
Mass-specific water loss at ~30% relative humidity. s – surviving individuals, d – dead individuals. [Measures: median ± first quartile and max/min (species names as in Figure 1)].

Under extreme dry conditions (RH ~30%) we found high mass-specific water loss at each investigated species (Figure 1). The specimens which died during the experiment lost significantly more water than the surviving ones (Figure 2). *Protracheoniscuspolitus* had the highest and *A.vulgare* had the lowest water loss (*P.politus* > *O.planum* > *C.convexus* > *T.rathkii* > *P.pruinosus* > *A.vulgare*). The ANOVA showed that *A.vulgare* lost significantly less water than the other species (p < 0.001; F value: 30.72). Mortality appeared in each species group except *A.vulgare* (*P.politus* – 100%, *P.pruinosus* – 80%, *C.convexus* – 65%, *O.planum* – 60%, *T.rathkii* – 25%).

At 75% relative humidity, the mass-specific water loss rates decreased in the order: *C.convexus* > *P.pruinosus* > *O.planum* > *P.politus* > *T.rathkii* > *A.vulgare* (Figure 1). Water loss differed significantly between *A.vulgare* and the other studied species groups (p < 0.001; F value: 30.98). Besides that, *T.rathkii* lost significantly less water than *C.convexus*, *O.planum* and *P.pruinosus*. Mortality was seen only in *P.pruinosus* (10%).

We detected the lowest water loss (Figure 1) without significant interspecific differences at the highest humidity level (~100%) (*C.convexus* > *T.rathkii* > *P.politus* > *A.vulgare* > *O.planum* > *P.pruinosus*). The mass-specific water loss showed negative correlation with the initial weight (correlation coefficient (R): -0.2663) and the tergal cuticle thickness (R: -0.3067). The thicker epi- (R: -0.2866) and procuticle (R: -0.3261) decreased the mass-specific water loss.

**Figure 3.**
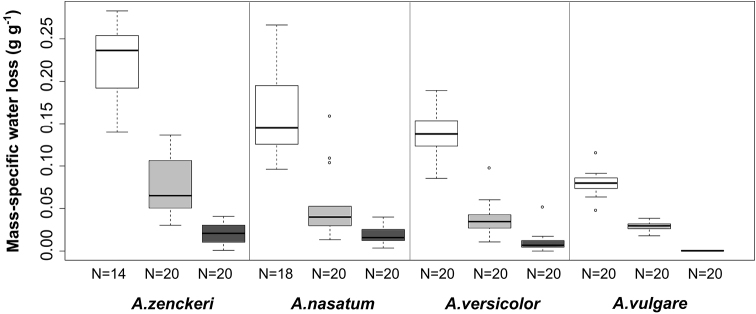
Mass-specific water loss of the survived individuals at the three different relative humidity values (white: ~30%, medium gray: ~75%, dark grey: ~100%). The experiment took 6 hours. [Measures: median ± first quartile and max/min; in species names *A.* means *Armadillidium*]

Within the genus *Armadillidium*, *A.vulgare* had the lowest and *A.zenckeri* had the highest water loss rates at 30% RH (Figure 3). Difference between *A.nasatum* and *A.versicolor* was not significant (p=0.16; F value: 23.21). Mortality occured in *A.zenckeri* (30%) and *A.nasatum* (10%).

Under higher humidity (75%) the order of mass-specific water loss was the same (Figure 3). *Armadillidiumzenckeri* lost significantly more water than *A.versicolor* and *A.vulgare* at this humidity (p < 0.001; F value: 7.793). At nearly 100% RH, the water loss rates were the lowest (Figure 3) but the ranking was the same (*A.zenckeri* > *A.nasatum* > *A.versicolor* > *A.vulgare*). Similarly to the interspecific study, within the *Armadillidium* genus, that is body size (R: -0.1020) and thicker cuticle (R: -0.3228) negatively correlated with mass-specific water loss rates. The mass-specific water loss showed negative correlation with the thickness of epi- (R: -0.3001) and procuticle (R: -0.3461).

The comparison of the desiccation resistance of the intrageneric and intergeneric groups has resulted in a lower F value for the former group, which means that there is a smaller relative variance among this group in comparison to the intergeneric group.

### Tergal cuticle thickness

**Figure 4.**
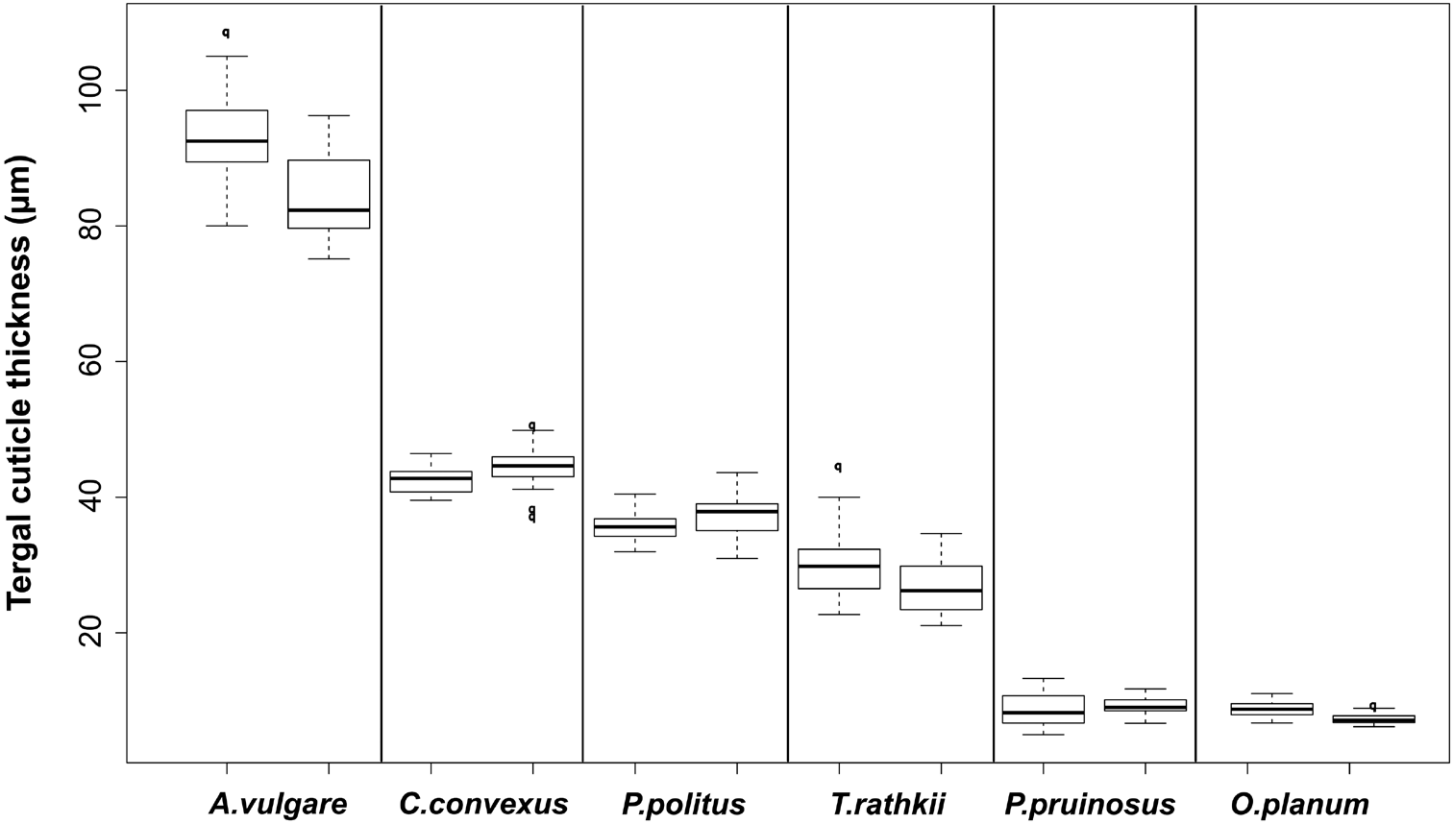
The tergal cuticle thickness in intra-, and interspecific relations (2 specimens/species, 5 slides/specimen, 10 measurements/slide). [Measures: median ± first quartile and max/min (species names as in Figure 1)].

Based on the average cuticle thickness the species can be sorted in decreasing order: *A.vulgare* > *C.convexus* > *P.politus* > *T.rathkii* > *P.pruinosus* > *O.planum* (Figure 4). Tergal cuticle thickness values were not significantly different between *P.pruinosus* and *O.planum* (p=1.000). For all species, thickness values were not significantly different intraspecifically (between the two specimens of the same species) (p > 0.59), but they differed significantly among species, that is interspecifically (p < 0.001).

**Figure 5.**
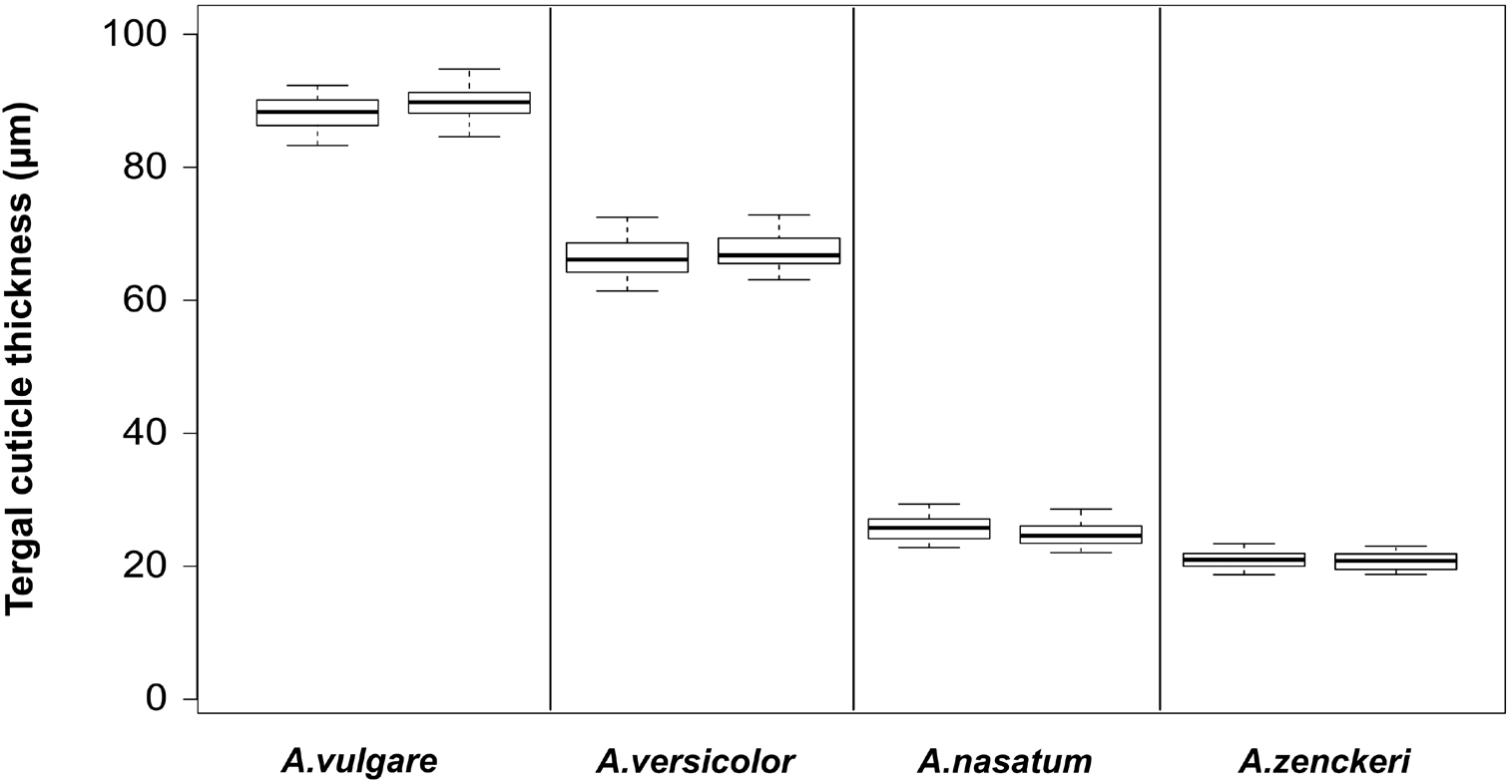
Intrageneric (between the two specimens per species) comparison of tergal cuticle thickness in four *Armadillidium* species (2 specimens/species, 5 slides/specimen, 10 measurements/slide). [Measures: median ± first quartile and max/min (Species names as in Figure 3)].

Within the *Armadillidium* genus, *A.vulgare* had the thickest tergal cuticle, while *A.zenckeri* had the thinnest cuticle (Figure 5). Our analysis revealed no significant difference intraspecifically (between the two specimens of the same species) (p > 0.69), but thickness differed significantly interspecifically (p < 0.001).

### Tergal surface structures

**Figure 6.**
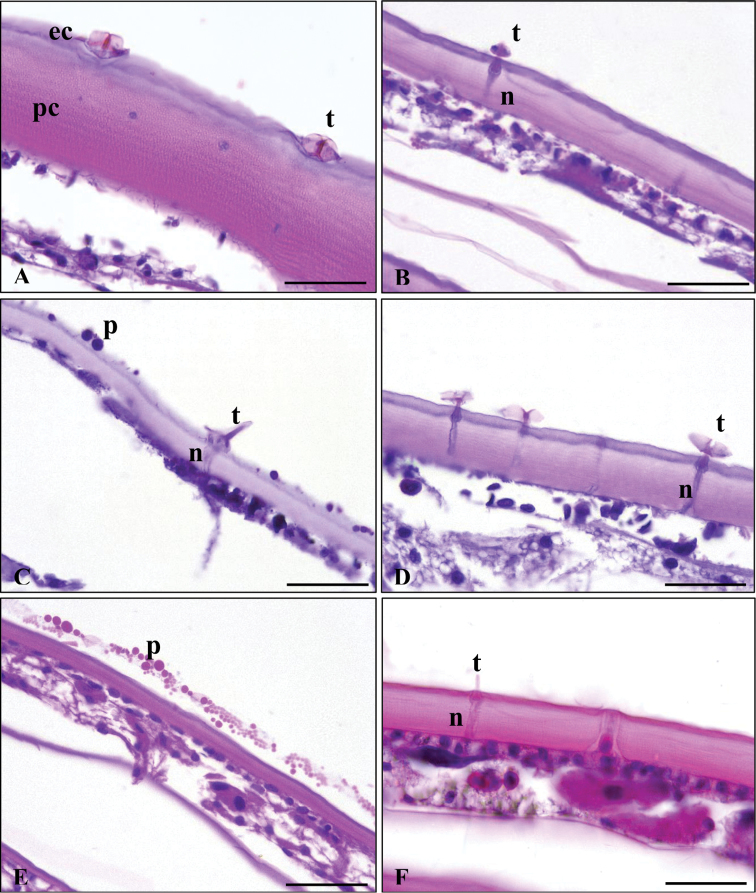
Light microscope micrographs of the studied species’ tergites. *Armadillidiumvulgare* (**A**), *Cylisticusconvexus* (**B**), *Orthometoponplanum* (**C**), *Protracheoniscuspolitus* (**D**), *Porcellionidespruinosus* (**E**), *Trachelipusrathkii* (**F**). Abbreviations: ec – epicuticle, pc – procuticle, p – polysaccharide spheres, t – tricorn receptor, n – nerve; x 63. Staining: hematoxylin-eosin (HE) – **A, E, F**; Periodic Acid-Schiff (PAS) – **B, C, D**. Scale bars: 50 µm.

**Figure 7.**
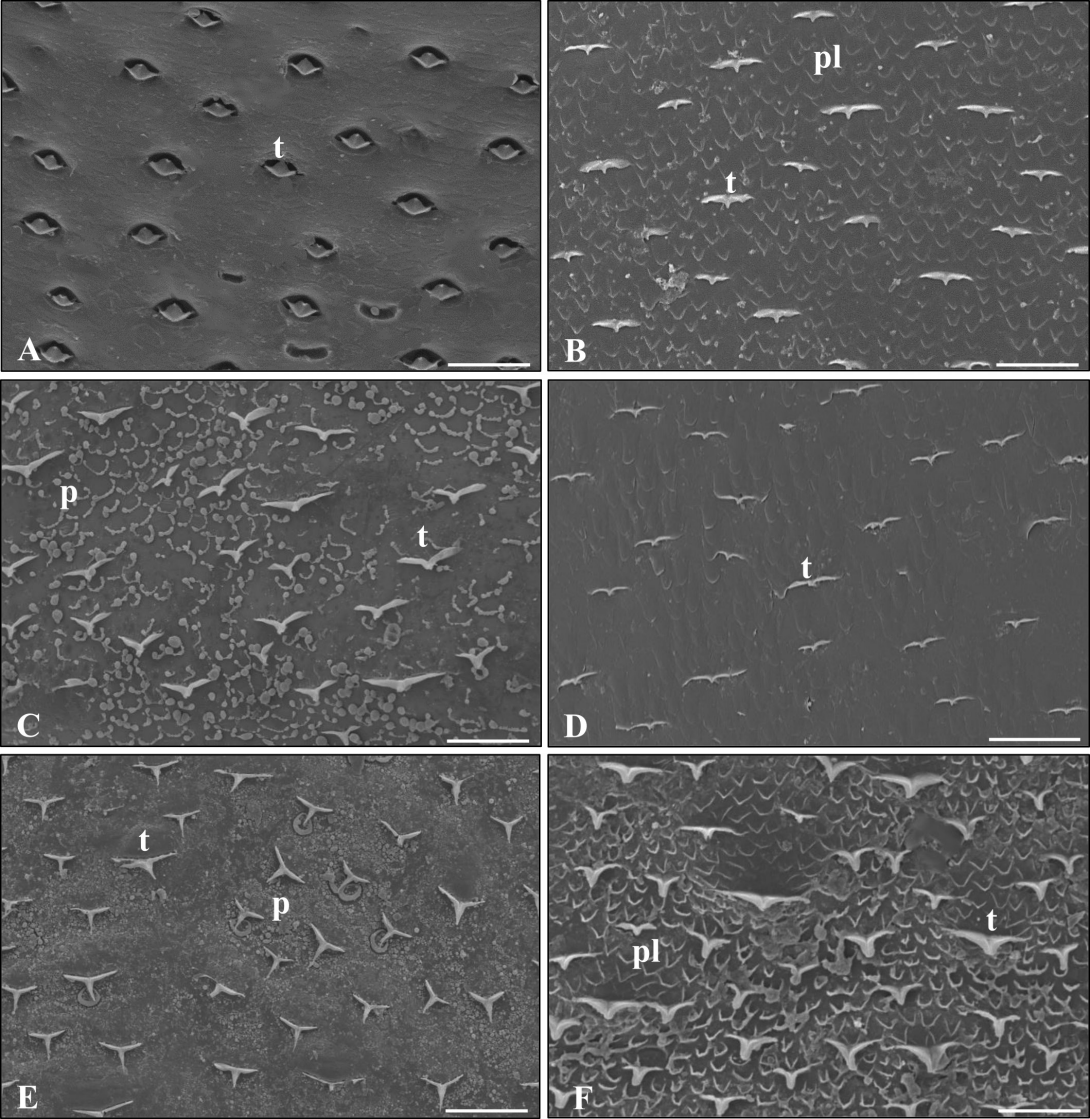
The dorsal surface of the studied sympatric terrestrial isopod species. *Armadillidiumvulgare* (**A**), *Cylisticusconvexus* (**B**), *Orthometoponplanum* (**C**), *Protracheoniscuspolitus* (**D**), *Porcellionidespruinosus* (**E**), *Trachelipusrathkii* (**F**). Abbreviations: pl – plaques, p – polysaccharide spheres, t – tricorn receptor. Scale bars: 50 µm.

**Figure 8.**
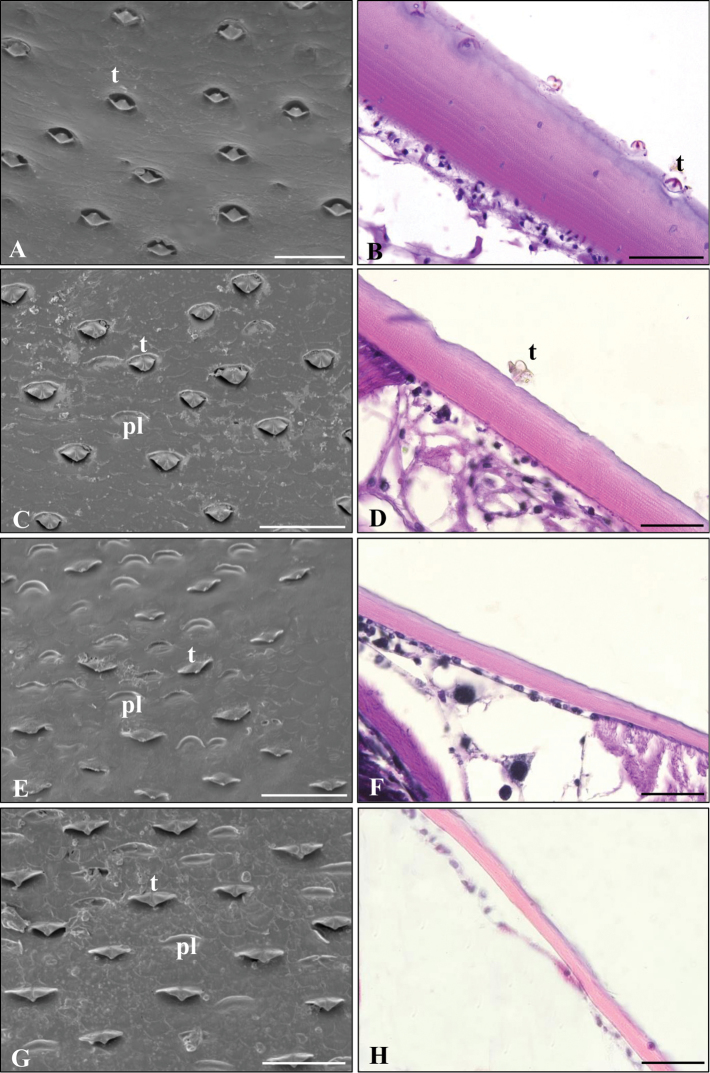
Scanning electron (**A, C, E, G**) and light microscope (**B, D, F, H**) micrographs on the studied *Armadillidium* species’ tergites. *Armadillidiumvulgare* (**A, B**), *A.versicolor* (**C, D**), *A.nasatum* (**E, F**), *A.zenckeri* (**G, H**). Abbreviations: pl – plaques, t – tricorn receptor. Staining: hematoxylin-eosin (HE) – **B, D, F, H**. Scale bars: 50 µm.

On the LM micrographs we found tricorn exteroreceptors in connection with neural processes (Figure 6B, C, D, F). The SEM micrographs showed variously shaped tricorns on the cuticular surface of each examined species (Figure 7). Closed, squat tricorns covered the surface of *A.vulgare* (Figures 7A, 8A) while *C.convexus*, *O.planum* and *P.politus* had elongated ones (Figure 7B–D). In the case of *O.planum*and *P.pruinosus*PAS-positive spheres covered the tergites (Figs 6C, E, 7C, E). The exoskeleton of *C.convexus* and *T.rathkii* was densely covered by plaques (Figure 7B, F).

Within the *Armadillidium* genus the SEM micrograph showed interspecific differences. There were squat tricorns on the tergites of *A.versicolor* (Figure 8C), but these were not as closed as in *A.vulgare* (Figure 8A). The tergal cuticle surface of *A.zenckeri* and *A.versicolor* were covered by similar tricorns and plaques (Figure 8).

## Discussion

In the present study we compared intergeneric (*A.vulgare*, *C.convexus*, *O.planum*, *P.politus*, *P.pruinosus*, *T.rathkii*) and intrageneric (*A.vulgare*, *A.versicolor*, *A.nasatum*, *A.zenckeri*) desiccation resistance of terrestrial isopods under three humidity ranges. As the applied experimental setup did not allow air to circulate, the calculated water loss might be underestimated. We assumed that differences in tolerance limits are connected to morphological characters such as tergal thickness and surface ornaments of the cuticle, which might be related to their habitat preferences.

Dias et al. (2012) showed that differences in body water loss rate serves as the main mechanism behind interspecific variation in mass-specific loss rate. Tsai et al. (1998) demonstrated on two *Ligia* species that intrageneric variation in desiccation resistance could be explained primarily by water loss rate, which is determined by body size. Body size affected desiccation resistance only indirectly, through changes in the water loss rate. Our results confirmed these previous findings: the larger initial weight decreased the mass-specific water loss rate at both intra- and intergeneric levels.

Several studies showed that body shape and cuticle permeability are also significant factors in water loss rate. Broly et al. (2015) studied the effect of surface area and mass on the desiccation rates in three different terrestrial isopod species: *Oniscusasellus*, *Porcellioscaber*, and *A.vulgare*. They found that the lower the individual surface area/volume ratio, the lower the individual water loss rate, both intra- and interspecific levels. According to their results body shape, rather than permeability, appears to explain the difference in mass-specific water loss rates between *A.vulgare* and *P.scaber*. In contrast, Warburg (1965) showed differences in permeability between isopod species with similar shape (*A.vulgare*, *Venezilloarizoniscus*).

In the present study individuals which died during the experiment lost more water than the surviving ones. Dias et al. (2012) observed that the water loss rate was nearly constant over time for all species both before and after the animals died. They suggested that water loss in terrestrial isopods is a passive process.

The cuticle of isopods is more permeable than that of the most terrestrial arthropods, and transpiration through the exoskeleton is a major part of water loss (Warburg 1993, Greenaway and Warburg 1998). Previous studies also suggested that the thickness of the exoskeleton was one of the morphological properties which determine the species’ distribution at different spatial scales (Csonka et al. 2013, Vittori and Štrus 2014). The experimental results also supported our expectations that the relatively thick cuticle offered an effective protection to the globally wide-spread, habitat generalist *A.vulgare*. We found that thicker epi- and procuticle separately also decreased the mass-specific water loss. The epicuticle has been proposed as a possible barrier against water loss by other authors (Cloudsley-Thompson 1977, Compère 1990).

Despite the relatively thick tergal cuticle *P.politus* did not survive under extreme dry conditions. The survival of oniscideans in natural habitats is critically dependent not only on habitat but also on daily activity patterns (Cloudsley-Thompson 1956, Edney 1968). According to Ilosvay (1982) and Tuf and Jeřábková (2008) *P.politus* is active mainly during twilight and early morning when humidity is higher, which minimizes desiccation. Activity peaks were at dusk and at midnight at a humidity of 75–80 %. In contrast, the common pill bug (*A.vulgare*) is active in the morning hours (Cloudsley-Thompson 1951), and very often also during the day (personal observations).

In the case of *O.planum* and *P.pruinosus* we found rather thin exoskeleton covered by polysaccharide spheres that might also reduce water loss. The composition and function of these structures is unknown. Hadley and Hendricks (1985) suggested that they were not composed of lipid as previously suggested (Ermin 1945, Schmalfuss 1978). According to Compère and Goffinet (1995) the glycoproteinaceous surface coat at *Carcinusmaenas* (Crustacea, Malacostraca, Brachyura) might be a hydrophilic layer protecting the cuticulin layer and/or reducing the surface tension between the hydrophobic cuticulin layer and the water. Tricorn receptors are common on the tergal surface, but according to Holdich (1984) there was a difference in their form in relation to the habitat. His results suggested that closed tricorns, as on the surface of *A.vulgare*, occur only in species living in drier environments. Besides these receptors, in some species (*C.convexus*, *T.rathkii*) we found plaques on the dorsal surface. According to Holdich (1984) these structures may be involved in the formation of tricorns.

Previous studies showed that ancestral terrestrial isopod species had lower desiccation resistance. Dias et al. (2012) suggested that the resistance against desiccation in Oniscidea differs among major phylogenetic groups. In accordance with this, the F values of our ANOVA analysis indicate smaller relative variance among the group means in intrageneric desiccation resistance.

Nevertheless, the desiccation resistance could not be explained by only phylogenetic relationship. This is further supported in the present study by the different water loss rate of *Armadillidium* species under dry conditions. Desiccation resistance of the four investigated species is in accordance with their cuticle thickness and habitat preference.

## Conclusions

Resistance against desiccation in terrestrial isopod species was significantly associated with the two investigated morphological traits: body mass (size) and thickness of tergal cuticle. Species with the smallest mass-specific water loss rate were larger and possessed thicker tergal cuticle. Significant variation in both desiccation resistance and morphological traits was observed among the four *Armadillidium* species, despite their close phylogenetic relatedness.

## Supplementary materials

Supplementary material 1 The non-significant results of the ANOVA tests Data type: (The non-significant results of the ANOVA test: Inter- and intrageneric comparisons of tergal cuticle thickness) This is an open access article distributed under the terms of the Creative Commons Attribution License (CC BY 4.0), which permits unrestricted use, distribution, and reproduction in any medium, provided the original author and source are credited.Diána Csonka, Katalin Halasy, Krisztina Buczkó, Elisabeth Hornung
