# Supplementary material for: Morphological traits – desiccation resistance – habitat characteristics: a possible key for distribution in woodlice (Isopoda, Oniscidea)
Source: Zookeys. 2018 Dec 3;(801):481–99. doi: 10.3897/zookeys.801.23088 (PMC6288246; doi:10.3897/zookeys.801.23088)
Supplement: Supplementary material 1 — The non-significant results of the ANOVA tests [file zookeys-801-481-s001.docx]

| **Inter-specific comparison** | **p-value (non-significant)** |
| --- | --- |
| *Porcellionides pruinosus1 - Orthometopon planum1* | 1.0000 |
| *Porcellionides pruinosus1 - Orthometopon planum2* | 0.0786 |
| *Porcellionides pruinosus2 - Orthometopon planum1* | 0.9999 |
| *Porcellionides pruinosus2 - Orthometopon planum2* | 1.0000 |
| **Intra-specific comparison** | **p-value (non-significant)** |
| *Armadillidium vulgare1 - Armadillidium vulgare2* | 1.0000 |
| *Cylisticus convexus1 - Cylisticus convexus2* | 0.5423 |
| *Orthometopon planum1 - Orthometopon planum2* | 0.0598 |
| *Porcellionides pruinosus1 - Porcellionides pruinosus2* | 0.9997 |
| *Protracheoniscus politus1 - Protracheoniscus politus2* | 0.8134 |
| *Trachelipus rathkii1 - Trachelipus rathkii2* | 0.7379 |

The non-significant results of the ANOVA test: Intergeneric comparison of tergal cuticle thickness

The non-significant results of the ANOVA test: Intrageneric comparison of tergal cuticle thickness

| **Intra-specific comparison** | **p-value (non-significant)** |
| --- | --- |
| *Armadillidium zenckeri1 – Armadillidium zenckeri2* | 0,707 |
| *Armadillidium nasatum1 - Armadillidium nasatum2* | 0,684 |
| *Armadillidium versicolor1 - Armadillidium versicolor2* | 0,758 |
| *Armadillidium vulgare1 - Armadillidium vulgare2* | 1,000 |
